# Direct activation of the fibroblast growth factor-21 pathway in overweight and obese cats

**DOI:** 10.3389/fvets.2023.1072680

**Published:** 2023-01-23

**Authors:** Emily J. Brinker, T. Jordan Towns, Rie Watanabe, Xiaolei Ma, Adil Bashir, Robert C. Cole, Xu Wang, Emily C. Graff

**Affiliations:** ^1^Department of Pathobiology, College of Veterinary Medicine, Auburn University, Auburn, AL, United States; ^2^Scott Ritchey Research Center, College of Veterinary Medicine, Auburn University, Auburn, AL, United States; ^3^School of Life Sciences and Technology, Tongji University, Shanghai, China; ^4^Department of Electrical and Computer Engineering, Samuel Ginn College of Engineering, Auburn University, Auburn, AL, United States; ^5^Department of Clinical Sciences, College of Veterinary Medicine, Auburn University, Auburn, AL, United States; ^6^Center for Advanced Science, Innovation and Commerce, Alabama Agricultural Experiment Station, Auburn, AL, United States; ^7^HudsonAlpha Institute for Biotechnology, Huntsville, AL, United States

**Keywords:** liver, weight loss, lipidoses, elasticity imaging techniques, proton magnetic resonance spectroscopy, gut microbiome

## Abstract

**Introduction:**

Feline obesity is common, afflicting ~25–40% of domestic cats. Obese cats are predisposed to many metabolic dyscrasias, such as insulin resistance, altered blood lipids, and feline hepatic lipidosis. Fibroblast Growth Factor-21 (FGF21) is an endocrine hormone that mediates the fat-liver axis, and in humans and animals, FGF21 can ameliorate insulin resistance, non-alcoholic fatty liver disease, and obesity. Activation of the FGF21 pathway may have therapeutic benefits for obese cats.

**Methods:**

In this preliminary cross-sectional study, *ad libitum* fed, purpose-bred, male-neutered, 6-year-old, obese and overweight cats were administered either 10 mg/kg/day of an FGF21 mimetic (FGF21; *n* = 4) or saline (control; *n* = 3) for 14 days. Body weight, food, and water intake were quantified daily during and 2 weeks following treatment. Changes in metabolic and liver parameters, intrahepatic triglyceride content, liver elasticity, and gut microbiota were evaluated.

**Results:**

Treatment with FGF21 resulted in significant weight loss (~5.93%) compared to control and a trend toward decreased intrahepatic triglyceride content. Cats treated with FGF21 had decreased serum alkaline phosphatase. No significant changes were noted in liver elasticity, serum, liver, or metabolic parameters, or gut microbiome composition.

**Discussion:**

In obese and overweight cats, activation of the FGF21 pathway can safely induce weight loss with trends to improve liver lipid content. This exploratory study is the first to evaluate the FGF21 pathway in cats. Manipulation of the FGF21 pathway has promising potential as a therapeutic for feline obesity. Further studies are needed to see if FGF21-pathway manipulation can be therapeutic for feline hepatic lipidosis.

## 1. Introduction

Obesity is prevalent in domestic cats and is associated with many co-morbidities, including insulin resistance and type 2 diabetes mellitus (T2DM) (1, 2). A significant sequela associated with feline obesity is increased intrahepatic lipid accumulation, predisposing cats to hepatic lipidosis (3, 4). The health risks associated with increased baseline liver lipid content are compounded by the mobilization of fatty acids from adipose tissue, increased insulin resistance, and the release of inflammatory mediators.

Fibroblast growth factor 21 (FGF21) is a member of the endocrine subfamily of fibroblast growth factors primarily produced in the liver that regulates the metabolic adaptation to fasting by binding to primarily FGF receptor 1c (FGFR1c) and its cognate co-receptor β-klotho in metabolic tissues (5). Among its numerous downstream effects, FGF21 stimulates gluconeogenesis, ketogenesis, and fatty acid oxidation in the liver and stimulates glucose uptake, lipolysis, and β-oxidation in the white adipose tissue (6). FGF21 analogs have been used in humans and animal models to ameliorate metabolic dyscrasias secondary to obesity. Studies that evaluate exogenous administration of FGF21 in obese and insulin-resistant humans show significant benefits such as weight loss, reduced blood glucose, reduced cholesterols and low-density lipoproteins, and resolution of hepatic steatosis with minimal to no side effects (7–9). LY2405319 (Eli Lilly and Company, Indianapolis, IN) is a modified human recombinant FGF21 analog engineered for increased stability and retained metabolic benefits and has been successfully used to treat obesity and hyperglycemia in rodent and primate models (10, 11). A benefit of LY2405319 is that it is more easily produced in large scales and has increased thermal and conformational stability over unmodified human recombinant FGF21 (12), meaning it can be prepared for a potential commercial, multiuse formulation suitable for veterinary hospitals. However, very little research has been done regarding FGF21 analog use in companion animals, and no research has been published on FGF21 in cats. Obese and overweight cats have an altered gut microbiota compared to lean cats (13), and common factors such as fasting have been shown to alter both circulating FGF21 and the gut microbiome (14, 15). While there is evidence that the microbiome regulates FGF21 (16), studies that evaluate the direct influence of exogenous FGF21 on the gut microbiome are limited (17).

Proton magnetic resonance spectroscopy (1H-MRS) is a non-invasive, highly sensitive and specific method of quantifying liver triglyceride content (18). 1H-MRS is commonly used for hepatic triglyceride quantification in studies of humans (19, 20) and many animal species (21, 22) and has been previously used to determine hepatic triglyceride content in lean and obese cats (4). Shear wave elastography methods are promising as a non-invasive tool for diagnosing and grading hepatic fibrosis and steatosis in humans (23, 24). Elastography has been safely used to evaluate the livers of healthy adult cats but has not been well-explored in veterinary medicine as a point-of-care diagnostic and monitoring modality for hepatic lipidoses (25).

The overall objective was to explore if FGF21 could be used safely and efficaciously to treat common obesity-associated metabolic dyscrasias, utilizing a research colony of obese and overweight cats with similar insulin resistance and metabolic alterations. Specifically, we focused on body weight, serum metabolic parameters, serum liver enzymes, and intrahepatic lipid content. Our primary hypothesis was that FGF21 administration would lead to decreased body weight independent of food intake. Following that, our secondary hypotheses were that FGF21 administration would improve glucose and lipid homeostasis, decrease intrahepatic lipid content, and potentially alter gut microbiota, secondary to weight loss. We also hypothesized that decreased liver lipid content would decrease hepatic elasticity and could potentially be used as a less invasive measure of hepatic lipid content in obese and overweight cats compared to a liver biopsy or 1H-MRS. In this preliminary study, we show that FGF21 significantly lowers body weight in cats. Additionally, FGF21-treated cats had a trend toward a decrease in hepatic triglyceride with a corresponding significant decrease in the enzymatic marker of hepatic triglyceride content, serum alkaline phosphatase (26). However, there were no detectable effects on lipid or glucose homeostasis or hepatic elasticity.

## 2. Materials and methods

### 2.1. Animals

Eight purpose-bred, specific pathogen-free, 6-year-old, neutered, Domestic Shorthair cat male cats were housed individually at the Scott Ritchey Research Center (Auburn University, AL). All studies were performed in line with the Auburn University Institutional Animal Care and Use Committee (IACUC) protocol (protocol number 2019-3482). Cats were provided environmental enrichment and allowed to group socialize under researchers' direct supervision once a day outside of their kennels. Animals were cared for according to the principles outlined by the United States Department of Agriculture (USDA) Animal and Plant Health Inspection Service's Animal Welfare Act and the American Associated for the Accreditation of Laboratory Animal Care (AAALAC) Guide for the Use of Laboratory Animals. No animals were euthanized for this study. All facilities are University IACUC approved, and USDA and AAALAC accredited.

Prior to the initiation of this study, cats were allowed to gain weight on a non-restricted chow diet formulated for cats (27.4% calories provided by fat, Feline Lab Diet, Purina, St. Louis, MO). This *ad libitum* diet was continued throughout the remainder of the study. At the start of the study, all cats were overweight or obese (defined as a body condition score of >5) and had evidence of insulin resistance but were not overtly diabetic or glucosuric. Based on comparisons from lean and obese body weight data and blood glucose levels, it was determined that a minimum of 3 cats for the treated and control groups was needed to get an effect size (β) of 0.8. At the start of the study, four cats were assigned to the treatment or control group (*n* = 4) to maintain no significant differences between blood glucose or body weight (unpaired *t*-test, *p* = 0.25 and *p* = 0.65, respectively) to conduct a cross-sectional study. After the initiation of saline injections, one of the control cats developed severe anorexia and was removed from the study on day 3 for medical reasons. Baseline blood glucoses and body weights remained non-significantly different after removal of the subject (*p* = 0.35 and *p* = 0.53, respectively).

### 2.2. Treatment

Cats were injected subcutaneously with a 10 mg/kg/day dose of sterile recombinant FGF21 (LY2405319) diluted with sterile saline to a final total daily injection volume of 5 mL or with 5 mL of sterile saline (control) for 14 days. Injection sites were rotated daily between the shoulder and hind limb and monitored for heat, redness, swelling, hair loss, or ulceration.

### 2.3. Weight, food, and water consumption measurements

Food and water consumption and body weight were determined daily. Each cat was allowed 200 g of dry food and 500 mL of water per day, and at no point did any cat consume all food or water given within 24 h.

### 2.4. Blood, urine, and feces collections

Before all blood, urine, and feces collections, cats were fasted for 10 h. Blood was collected from cats under general anesthesia or sedation. The week before injections and on day 14 of treatment, a complete blood count (Advia 120 Hematology, Siemens), standard serum biochemistry panel with triglycerides (Cobas C 311 Analyzer, Roche), blood glucose (AlphaTrak2) (27), and a urinalysis (Multistix 10 SG, Siemens) was performed on each cat. Urine was collected either free catch with manual expression or with ultrasound-guided cystocentesis. Serum was separated from the remaining whole blood through centrifugation at 800 g for a minimum of 15 min (Heraeus Megafuge 16R, Thermo Scientific) and stored at −80°C until needed. Non-Esterified Fatty Acids (NEFAs) and insulin were quantified from serum using the previously validated and commercially available HR Series NEFA-HR (2) (Wako Diagnostics) (27) and Feline Insulin ELISA (Mercodia) kits (28), respectively. Feces were collected by placing a plastic fecal loop into the rectum and descending colon to obtain an adequate amount (>200 mg) of feces. All fecal specimens were stored until analysis at −80°C. The homeostatic model assessment for insulin resistance (HOMA-IR) was calculated as the product of the basal glucose and insulin concentrations, divided by 22.5, as previously described and validated in the literature (29, 30). Adipose tissue insulin resistance (Adipo-IR) was calculated as the product of fasting serum insulin and NEFAs (31).

### 2.5. Whole genome shotgun metagenomic sequencing of the fecal microbiome

Two fecal samples were collected from each cat using a fecal loop with and without lubrication (*N* = 14 total; Supplementary Table S1) (32). At least 200 mg feces were used for DNA extraction by Qiagen Allprep PowerFecal DNA/RNA kit (Qiagen, MD), following the protocols provided by the manufacturer. During DNA extraction, fecal samples were homogenized by the Qiagen PowerLyzer24 instrument (Qiagen, MD) to achieve homogeneous results. The extracted DNA concentrations were measured by the Qubit 3 Fluorometer (Invitrogen, CA), and A260/A280 absorption ratios were assessed using the NanoDrop One C Microvolume Spectrophotometer (Thermo Fisher Scientific, MA). DNA fragmentation was performed by M220 Focused-ultrasonicator (Covaris, MA) on 1.5–2 μg of DNA for each sample to achieve fragmented DNA of ~500 bp. NEBNext Ultra II DNA Library Prep Kit for Illumina (New England Biolabs, MA) was used to construct WGS metagenomic sequencing libraries using the fragmented DNA. Final library concentrations and size distributions were measured by LabChip GX Touch HT Nucleic Acid Analyzer (PerkinElmer, MA) before being sequenced on an Illumina NovaSeq6000 sequencing machine on the 150-bp paired-end mode at the Genomics Service Laboratory at the HudsonAlpha Institute for Biotechnology (Huntsville, AL).

### 2.6. Metagenomic data analysis

Adapter sequences and low-quality sequences were eliminated with Trimmomatic (v0.36) (33). Filtered reads were then mapped to the cat reference genome (GCF_000181335.3), viral genome database, and rDNA sequences downloaded from National Center for Biotechnology Information (NCBI) using Burrows-Wheeler Aligner (BWA) (v0.7.17–r1188) (34) and SAMtools (v1.6) (35). The remaining microbial reads were extracted using BEDTools (v2.30.0) and aligned to the feline gut microbiome reference contigs (GCA_022675345.1) (13). The alpha- and beta-diversity of taxonomy profiles were performed using R package vegan v2.5.7 (36) at the species level using Shannon index and Bray-Curtis dissimilarity. Mann Whitney U Tests (37) and permutational multivariate analysis of variance (PERMANOVA) (38) were used to determine significant differences in alpha- and beta-diversities between FGF21 and saline-treated cat groups. To assess the statistical significance of the differential abundance of species between treatments, Kruskal–Wallis tests (39) were performed in R. The adjusted P-values were calculated using R package qvalue (v2.22.0) (40). The criteria for detecting significantly alter microbial species are qvalue < 0.05 and log2 fold change > 1.5.

### 2.7. Calculation of lipid fraction in liver

Within 1 week prior to the start of FGF21 injections, cats were anesthetized and proton magnetic resonance spectroscopy (1H-MRS) using a Siemens Magnetom 7T Actively-Shielded Scanner was performed to determine the lipid fraction of the liver. 1H-MRS was repeated on day 14 of the FGF21 or saline injections.

Cats were positioned in ventral recumbency, and bellows were used for respiratory gating. The knee coil was used in signal acquisition. Three plane respiration-guided scout images were acquired for spectroscopy volume localization, and STEAM pulse sequence was used for MRS data acquisition. Data was acquired from 5 × 5 × 5 mm^3^ spectroscopic volume placed in the right hepatic lobe. Animals were allowed to breathe freely throughout the scan while ensuring data were collected from the specified voxel location in the liver *via* bellows-based respiratory gating. Data were acquired with TE = 8 ms, TR = 6 s, TM = 20 ms, and 32 averages were collected. The spectral width was 3,200 Hz, and the vector size was 2,048 points.

Spectra were analyzed using the AMARES module of jMRUI (41, 42). Briefly, water (H_2_O at 4.7 ppm), methyl (CH_3_ at 1.3 ppm), and methylene (CH_2_ at 0.9 ppm) resonances were modeled with Lorenzian sinusoids to determine the area under the resonances. The fat fraction was defined as the ratio of the areas of the methyl and methylene resonances to that of the water plus methyl and methylene (4, 43). The average value of the fat fraction was determined from 2 unique liver voxels.

### 2.8. Calculation of organ elasticity

The use of 2D shear wave elastography with acoustic radiation impulse force (ARIF) technology is used to assess tissue elasticity (44). Elastography has been established in the livers of healthy cats (25) and is primarily used to evaluate feline chronic kidney disease (45).

Shear wave elastography was performed using a Toshiba Aplio 500 ultrasound machine with a 15L5 linear probe before and after the injection period under heavy sedation. Tissue elasticity value was recorded using shear wave speed quantified as Young's modulus and expressed in kPa. Between 6 and 8 intralobular sites within the right liver, central liver, and left liver were acquired with the tissue elasticity reported from the average across the three portions (right, central, left) of the liver. Areas within the liver devoid of large vascular or ductal structures were chosen. The shear wave sampling ROI and depth were kept uniform between patients.

### 2.9. Statistical analysis

Metabolic parameters, liver lipid content, and elastography are all presented in box and whisker plots as individual animals change from baseline in both the FGF-treated and control groups. Whiskers on box and whisker plots represent minimum and maximum values. Unpaired student *t*-tests were performed to compare individual changes between FGF21 and control groups. Paired *t*-tests were performed for all comparisons within the FGF21 and saline groups.

Changes from baseline of weight, food, and water intake were calculated in a repeated measures fashion to account for individual variability. Water and food consumption were calculated as a ratio to body weight for each cat as mL/kg (“corrected water intake”) or g/kg (“corrected food intake)” to control for differences in water and food intake that may be due to differences in body weight.

To determine the rate of weight loss and weight gain, the slope was obtained from simple linear regressions on the percentage weight change from baseline to the nadir weight (weight loss) and then from nadir weight to end of study weight (weight gain). All areas under the curve (AUC) represent net AUC with inclusion of peaks below baseline and no minimum peak height and were calculated using the linear trapezoidal method. AUCs were compared by using unpaired *t*-tests.

For the supplemental data representation of metabolic, liver lipid content and elastography, absolute values at baseline and end point are plotted in bar graphs with individual animals denoted. Data in Supplementary Figures represent mean ± SD. Paired *t*-tests were performed for all comparisons within the FGF21 and control groups, and unpaired *t*-tests were performed for all comparisons between the FGF21 and control groups.

All statistical methods except those used for microbiome analysis in the metagenomic data analysis section were performed using GraphPad Prism 9 (La Jolla, CA). An alpha level of 0.05 was used to determine statistical significance for all methods.

## 3. Results

### 3.1. Morphometric Parameters

**Figure 1:**
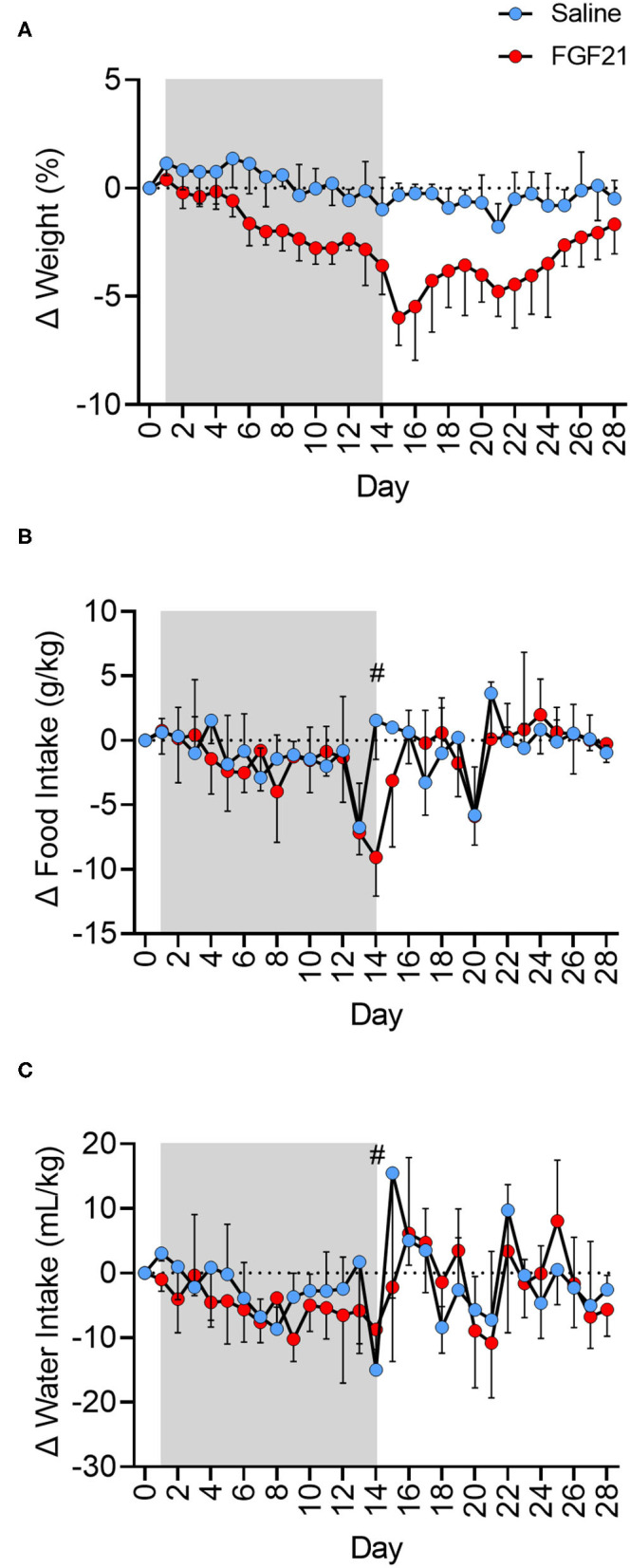
FGF21 reduces body weight independent of changes in food or water intake. **(A)** Treatment with FGF21 resulted in a significant decrease in body weight at a rate 2.54 times faster than in saline-treated cats. On day 15, FGF21-treated cats had a 5.93% decrease in body weight, with only a 0.28% decrease in body weight in saline-treated cats. Discontinuation of FGF21 resulted in a rapid regain of weight. **(B)** For the FGF21-treated group, the net AUC of corrected food intake from baseline for the treatment period was −26.75 g^*^day/kg (±13.1, 95% CI). The net AUC of corrected food intake for this same treatment period for the saline-treated group was similar at −17.08 g^*^day/kg (±12.6, 95% CI). Additionally, the FGF21-treated and saline-treated groups ate a similar amount of food during the washout period (*p* = 0.94). For the FGF21-treated group, the net AUC of corrected food intake from baseline for the washout period was −4.02 g^*^day/kg (±13.6, 95% CI). The net AUC of corrected food intake for this same washout period for the saline-treated group was similar at −4.78 g^*^day/kg (±11.6, 95% CI). **(C)** The net AUC for corrected water intake from baseline for Days 1 to 28 of the study for the FGF21 treatment was −82.93 mL^*^day/kg (±42.7, 95% CI), and the net AUC for corrected water intake for the same period for the saline-treated group was −46.27 mL^*^day/kg (±47.5, 95% CI). The FGF21-treated cats drank a similar amount of water during the treatment period (Days 1 to 14) as the saline cats (*p* = 0.10). During the treatment period for the FGF21-treated cats, the net AUC for corrected water intake was −68.12 mL^*^day/kg (±26.8, 95% CI). For the same period, the net AUC for corrected water intake for the saline-treated cats was −35.68 mL^*^day/kg (±23.3, 95% CI). Both groups had similar levels of water intake during the washout period, or from day 15 to 28 (*p* = 0.95). For the washout period, the net AUC of corrected water intake for the FGF21 treated cats was −9.358 mL^*^day/kg (±30.8, 95% CI), and the net AUC of correct water intake for the control cats was −10.84 mL^*^day/kg (±40.2, 95% CI). Each data point of food and water intake represents 24 hours of intake. On day 14 (#), cats were held without food overnight in preparation for general anesthesia and 1H-MRS data. Shaded areas between days 1 and 14 represent the period where either FGF21 or saline vehicle were given.

FGF21-treated cats had a steady decrease in body weight percentage at a rate 2.54 times greater than the control cats (Figure 1A, *p* < 0.0001). The mean weight loss from baseline of the FGF21-treated cats reached a maximum of 0.375 kg on day 15 post initial FGF21 injection (Supplementary Figure S1A, Supplementary Table S2), corresponding to a 5.93% decrease in body weight. At the same point in the study (Day 15), the saline-treated group had only a mean weight loss of 0.017 kg in body weight or a 0.28% decrease in body weight. During the treatment period (Day 1 to Day 15), the rate of body weight loss for FGF21 treated cats was 0.34% (±0.062, 95% confidence interval [CI], Y = −0.3395^*^X + 0.7709, *R*^2^ = 0.68) compared to 0.13% (±0.071, 95% CI, Y = −0.1339^*^X + 1.407, *R*^2^ = 0.25) for control cats. During the two-week washout phase, or days 15 to 28, FGF21-treated cats began to regain weight. Each day without FGF21 treatment corresponded to a 0.265% (±0.11, 95% CI) regain in weight compared to Day 15 (Y = 0.2653^*^X−9.452, *R*^2^ = 0.30). The net AUC for the weight change for days 1 to 28 for FGF-treated cats was −4.6 kg^*^day (±0.61, 95% CI), whereas the net AUC for this same period for the saline-treated cats was −0.13 kg^*^day (±0.44, 95% CI, *p* < 0.0001).

The FGF21-treated and control groups had similar food intake for the entire study period, or days 1 to 28 (*p* = 0.26, Figure 1B, Supplementary Figure S1B). For the FGF21-treated group, the net AUC for corrected food intake from baseline between days 1 and 28 was −36.86 g^*^day/kg (±19.8, 95% CI). For the control group, the net AUC for corrected food intake for the same period was−20.58 g^*^day/kg (±17.4 g, 95% CI). When examined at different times during the study, the FGF21-treated and control groups also ate a similar amount of food during the treatment period (day 1 to 14) (*p* = 0.11) as well as during the washout period (day 15 to 28) (*p* = 0.94).

Both groups had similar water intake throughout the study period (*p* = 0.27, Figure 1C, Supplementary Figure S1C).

### 3.2. Metabolic parameters

**Figure 2:**
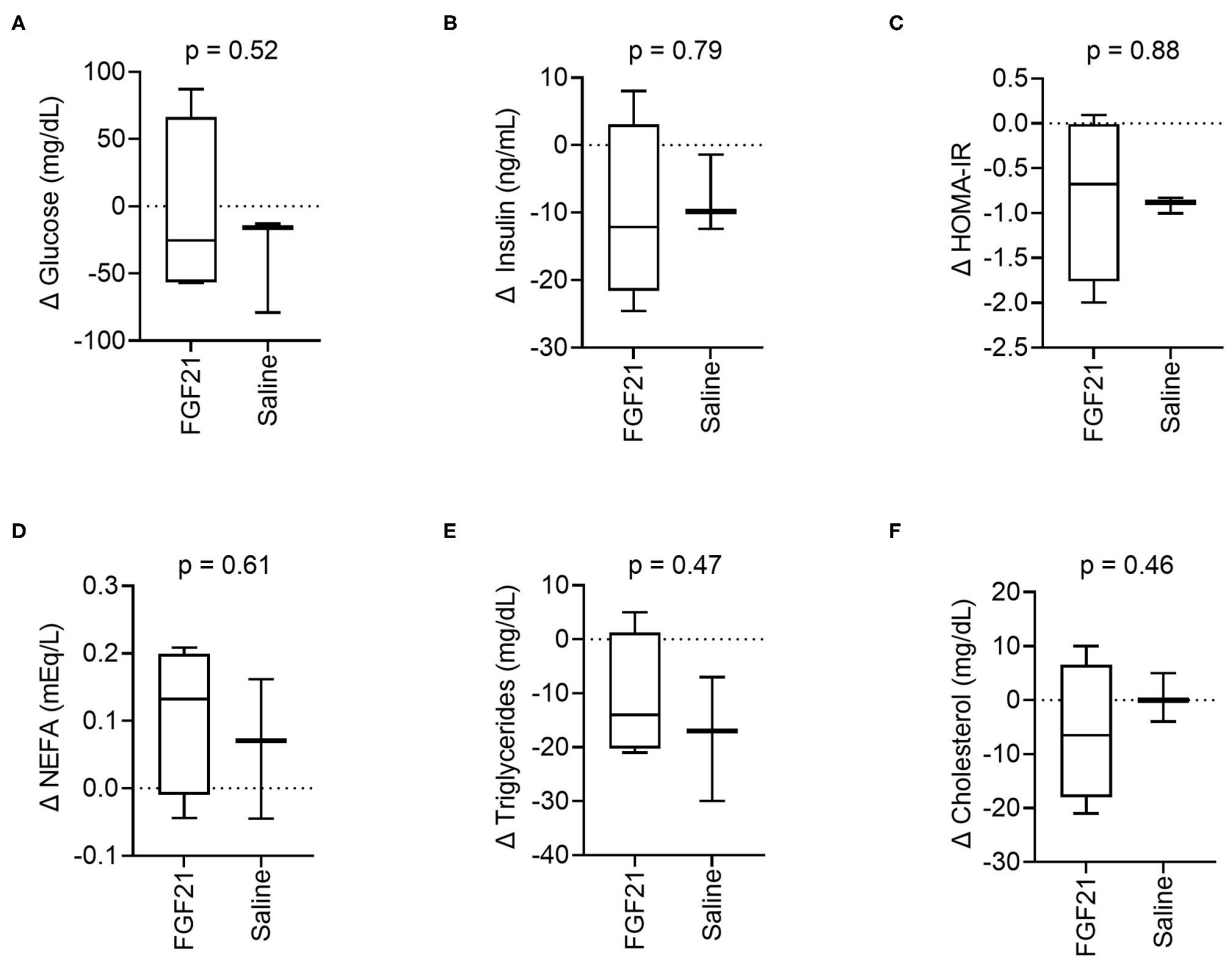
Circulating metabolic parameters. **(A)** Treatment with FGF21 did not alter blood glucose differently than saline (unpaired *t*-test). Similar findings were noted with **(B)** insulin, **(C)** insulin resistance, **(D)** non-esterified fatty acids, **(E)** triglycerides, and **(F)** cholesterol.

At the start of the study, baseline blood glucose was similar between treatment groups (*p* = 0.35, Supplementary Figure S2A). After 14 days of treatment, the changes in blood glucose between the FGF21-treated and control groups were also similar (*p* = 0.52, Figure 2A). In both groups, absolute blood glucose concentrations remained steady over the treatment period with no significant changes noted (FGF21; *p* = 0.89 and control; *p* = 0.24, Supplementary Figure S2A).

The serum insulin levels were similar at the beginning of the study between the FGF21-treated and control groups (*p* = 0.74, Supplementary Figure S2B). There was no significant difference in the change in serum insulin concentrations between the FGFf21 and control groups (*p* = 0.79, Figure 2B) following the 14-day treatment period. At the end of the treatment period (Day 14), the groups had similar serum insulin levels with no significant difference detected (*p* = 0.93) (Supplementary Figure S2B).

In cats, HOMA-IR is considered a predictor of insulin resistance (30) and correlates with body fat (29). FGF21 treatment did not significantly alter changes in insulin resistance, and the change in HOMA-IR between the groups was not significantly different following the treatment period (*p* = 0.88, Figure 2C), consistent with both insulin and glucose. However, the control group decreased insulin resistance between days 0 and 14 (Supplementary Figure S2C). As with blood glucose and serum insulin, HOMA-IR between the FGF21-treated and control groups were similar at the beginning of the study (*p* = 0.10, Supplementary Figure S2C).

FGF21 treatment did not significantly affect changes in serum NEFAs (*p* = 0.61, Figure 2D), triglycerides (*p* = 0.47, Figure 2E) or cholesterol (*p* = 0.46, Figure 2F) concentrations in cats. The NEFA, triglyceride, and cholesterol levels were similar at the beginning of the study for both the FGF21-treated and control groups (*p* = 0.76 and *p* = 0.26 and *p* = 0.83, respectively). Concentrations of the lipid parameters did not change within groups throughout the study (Supplementary Figures S2D–F).

Adipo-IR is used in humans and rodents as a non-invasive measure of insulin resistance in adipose tissues. FGF21 did not significantly alter changes in Adipo-IR between the FGF-21 and control groups (*p* = 0.97, Supplementary Figure S2G). In both groups, Adipo-IR remained steady for the treatment period with no significant changes noted (FGF21; *p* = 0.98 and control; *p* = 0.95, Supplementary Figure S2H).

### 3.3. Assessment of hepatic triglyceride content and elasticity

**Figure 3:**
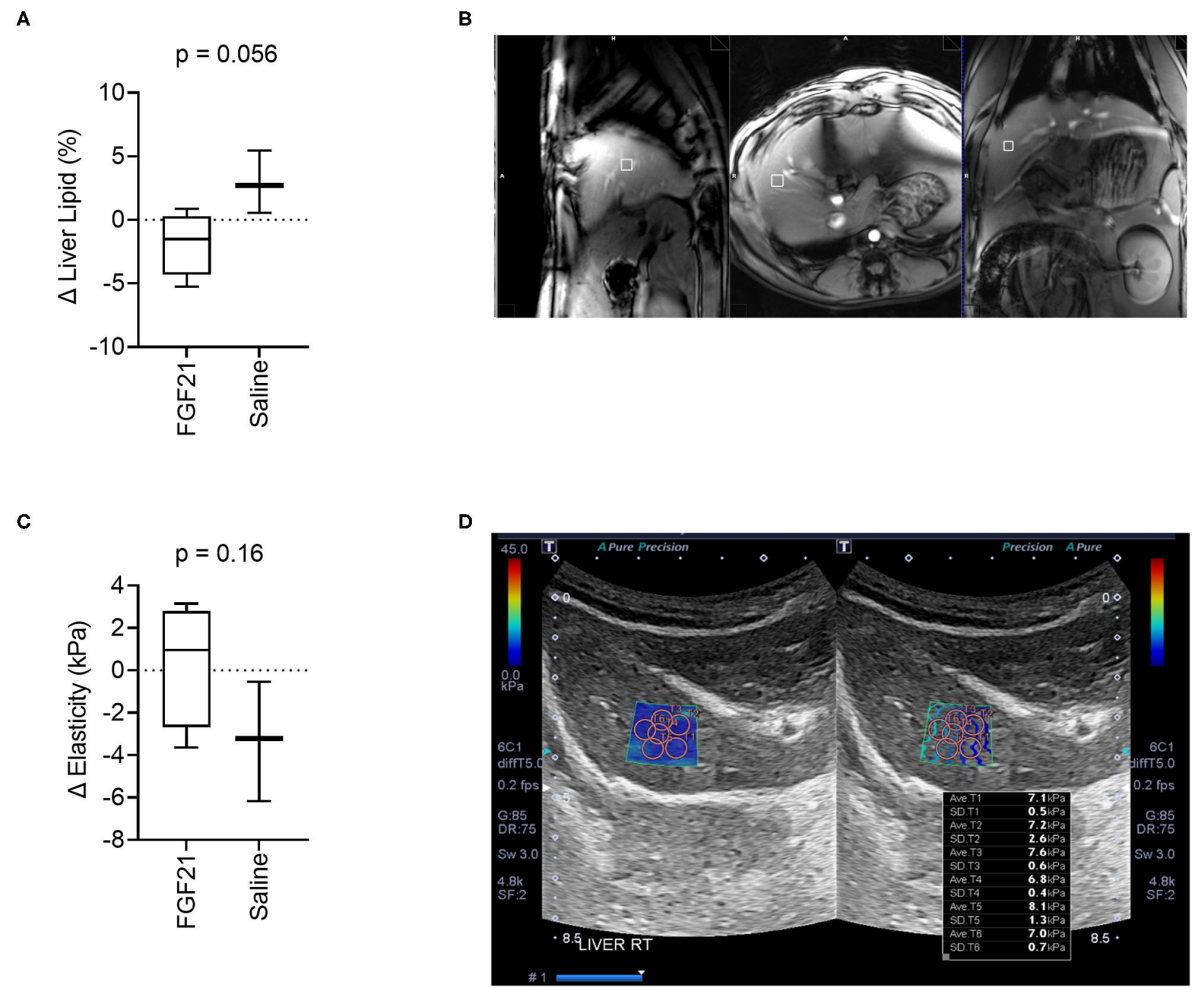
FGF21 tends to protect from liver lipid accumulation but does not alter liver tissue stiffness. **(A)** The fraction liver lipid measured by 1H-MRS is not significantly altered by FGF21 but tends to decrease compared to lipid alterations from saline treatment (unpaired *t*-test). **(B)** Example of voxel selection for 1H-MRS from a control cat. The white box delineates the voxel region**. (C)** Liver tissue stiffness is not altered by FGF21 treatment (unpaired *t*-test). **(D)** Example of measurements of stiffness using elastography of a single hepatic lobe from a saline-treated cat.

Assessment of lipid fractions based on H1-MRS of regions from the right hepatic lobe indicates a trend toward decreased liver lipid content in cats treated with FGF21 compared to control (Figures 3A, B); however, the change in liver triglyceride content between the two treatment groups did not reach statistical significance (*p* = 0.055, Figures 3A, B). As expected for obese and overweight cats and consistent with other studies (4), the baseline values for liver triglyceride content before treatment averaged 4.79 ± 3.36% SD, and there was no difference in liver lipid content between groups at the start of the study (*p* = 0.10). Despite significant and rapid weight loss, FGF21-treated cats had a 1.86% decrease in liver triglyceride content (Supplementary Figure S3A). In contrast, saline-treated cats had a 2.89% increase in liver triglyceride content.

Liver tissue elasticity was evaluated using 2D shear wave elastography with acoustic radiation impulse force (ARIF) (Figure 3D). There was no difference in change in liver elasticity between treatment and controls (*p* = 0.16, Figures 3C, D).

### 3.4. Serum hepatic analytes

**Figure 4:**
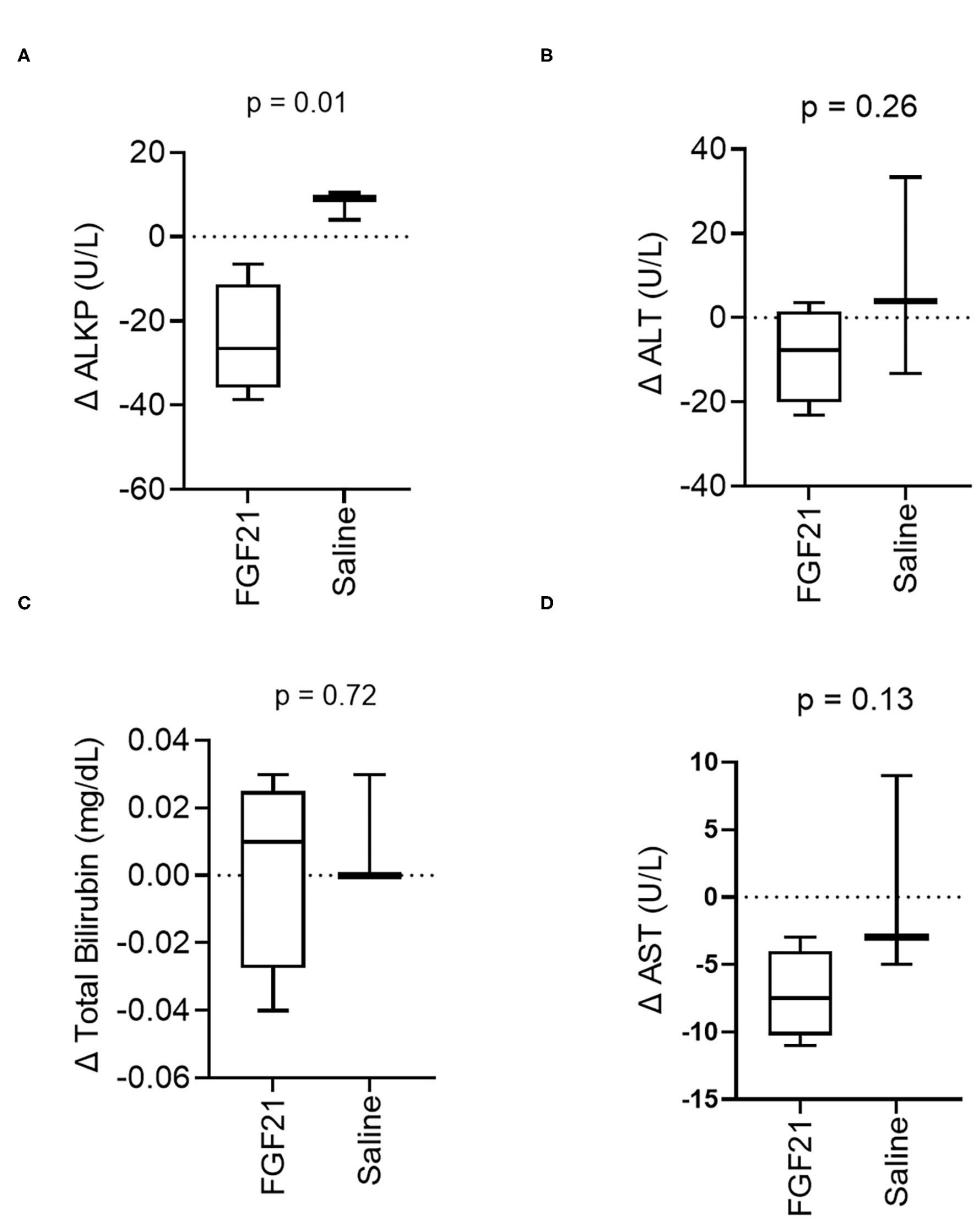
Hepatic serum biomarker changes are consistent with decreased liver lipid content. **(A)** ALKP activity, an inducible liver enzyme and a sensitive indicator of hepatic lipidosis in cats, overall significantly decreases with FGF21 treatment (unpaired *t*-test). **(B)** ALT, **(C)** Total Bilirubin, and **(D)** AST are not altered by FGF21 treatment when compared to alterations by saline.

A significant difference was noted in the change in alkaline phosphatase (ALKP) activity between treatment groups (*p* = 0.01, Figure 4A, Supplementary Figure S4A), a marker of feline hepatic lipidosis (26). There were no significant differences in the changes in serum alanine aminotransferase (ALT) (*p* = 0.26, Figure 4B, Supplementary Figure S4B) or changes in total bilirubin (*p* = 0.72) (Figure 4C, Supplementary Figure S4C) between control and FGF21 treatment. The individual changes in AST in FGF21 and control groups were similar (*p* = 0.13, Figure 4D).

### 3.5. Gut microbiota

**Figure 5:**
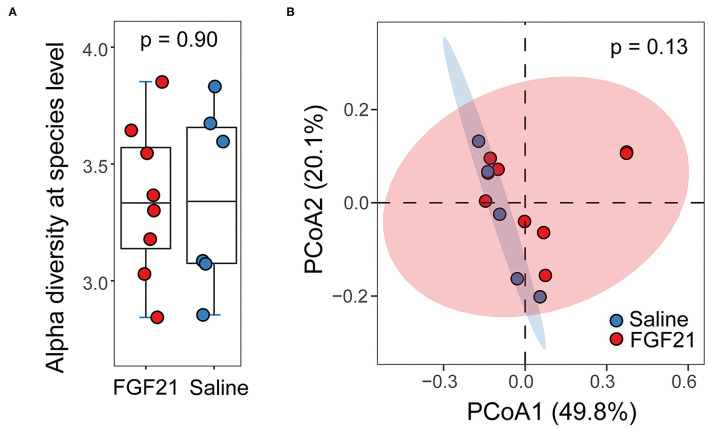
FGF21 treatment did not alter the feline gut microbiome. **(A)** Boxplots of alpha diversity in cat gut microbiome of FGF21-treated (red) and saline-treated (blue) groups at the species level measured by the Shannon index. **(B)** The Principal Coordinates Analysis (PCoA) plots of beta diversity between FGF21-treated (red) and saline-treated (blue) groups using Bray-Curtis distance. Statistical significance was assessed using permutational multivariate analysis of variance (PERMANOVA).

A total of 168 Gb metagenomic sequences were obtained for the 14 fecal microbiomes, with an average of 79.9 million reads per metagenome (Supplementary Table S1). The average host contamination is 10.6%. Taxonomic annotation and relative abundance quantification were performed according to a pipeline reported previously in Ma et al. (13). A total of 8,582 bacterial species were identified in the 14 cat metagenomes. Between the FGF21-treated and control saline-treated group, no significant difference in microbial alpha diversity was discovered (*p* = 0.90, Kruskal-Wallis rank sum test; Figure 5A). The Principal Coordinates Analysis (PCoA) plot of beta diversity did not reveal any significant separation of the two treatment groups either (*p* = 0.13, PERMANOVA, Figure 5B). At an FDR < 5% and minimum log2 fold change of 1.5, none of the microbial species have significant differences in abundance between FGF21 and saline-treated groups, suggesting a lack of changes in the gut microbiome.

### 3.6. Safety

There was no evidence of tissue necrosis or sloughing in the FGF21-treated and control cats at the injection sites. Some mild pain with palpation was noted at the injection site in both groups. All cats in the study remained healthy, apart from obesity, within a 6-month post-treatment observation period. All cats treated with FGF21 are alive and reportedly in good health at 2 years post-treatment follow-up.

## 4. Discussion

This is the first study to describe the effects of activation of the novel FGF21 pathway in cats. This study provides strong evidence that FGF21 analogs can safely induce weight loss in obese and overweight cats without major influence on caloric (food) intake or water intake. Subcutaneous injections of 10 mg/kg/day of the FGF21 analog LY2405319 in obese and overweight cats safely, steadily, and significantly decreased their weight, despite *ad libitum* feeding and without changes in food intake, consistent with observations in many studies of FGF21 administration in rodents, non-human primates, and humans (9, 11, 46–49). Once FGF21 was discontinued in the cats, we observed an immediate and steady return to baseline body weight, similar to what was noted during the post-treatment period in non-human primates (11, 46, 50). The decrease in weight without a concurrent reduction in caloric intake points to increased energy expenditure *via* the basal metabolic rate modulation as reported in rodents (10, 51, 52). Mice treated with recombinant FGF21 have dose-dependent weight loss from increased resting energy expenditure and fat utilization without decreasing food intake (10, 51, 52). In non-human primates, many publications describe weight loss concurrent with reduced food intake, but whether or not the weight loss was solely due to decreased caloric intake is debated (11, 46, 48, 49). Other causes of weight loss without reduced caloric intake, rather than increased basal metabolic rate, such as decreased nutrient absorption, are unlikely as there were no supportive clinical signs such as diarrhea or steatorrhea. In addition, the absence of significant differences between control and FGF21-treated cats in the gut microbiome suggests GI malabsorption is unlikely. Future studies investigating FGF21 in cats should evaluate metabolic mechanisms for weight loss, including fat distribution and body composition, appetite, and resting metabolic rate determination. The significant decrease in body weight is somewhat surprising given the preliminary nature of this study and the low numbers of animals in each group. These findings indicate that the dose was sufficient to elicit an effect in obese and overweight cats.

Contrary to our expectations, we did not see changes in circulating glucose, insulin, or lipid parameters (NEFAs, cholesterol, triglycerides). HOMA-IR has been used as an indicator of insulin sensitivity in cats, and the overweight and obese cats used in this study had HOMA-IR calculations consistent with reported values for obese and overweight glucose intolerance (30). Additionally, overweight and obese cats with increased proportions of body fat have greater insulin resistance as calculated with HOMA-IR (29). We expected that FGF21 treatment would improve insulin sensitivity as reported in other species, but the overall decreases in insulin sensitivity remained static between the control and FGF21-treated groups. Adipo-IR is a non-invasive predictor of specifically adipose tissue insulin resistance (31, 53). Our study did not show any alterations in Adipo-IR with FGF21 treatment, despite the weight loss. This suggests that FGF21 drives lipolysis of the adipose tissue stores without a change in adipose insulin resistance. The predictive measure Adipo-IR has not previously been used in cats, so further studies are needed to determine how well this value translates from humans and rodent models. One explanation for these findings is that none of the cats used in this study had marked metabolic dyscrasia or type 2 diabetes mellitus (T2DM), unlike the human and non-human primates in other studies treated with FGF21. Potentially, insulin resistance and dyslipidemia were not severe enough in our cats for FGF21 pathway activation to significantly affect the treatment group. A lack of FGF21 response in more metabolically healthy animals has been reported in Siberian hamsters in a study where the leaner animals had a reduced to absent effect of FGF21 on glucose homeostasis and weight loss (54). Another possibility is that the chow diet blunted the FGF21 response. Supporting this, non-obese chow-fed mice treated with FGF21 have blunted increase in energy expenditure than high-fat diet mice (55). These factors, in combination with the few subjects in each cohort, limited the power of the study to detect changes in these parameters. Further studies are warranted in cats with more severe metabolic disease to determine if FGF21 treatment indeed lacks influence on this species.

In rodent models, treatment with FGF21 has beneficial metabolic effects on adipose tissue including adipose tissue browning and thermogenesis, increased insulin sensitivity of adipose tissue, a reduction of adipose tissue macrophages, promotion of adipose glucose disposal, and lipolysis of white adipose tissues. These mechanisms have a compounding effect on increasing total energy expenditure, lowering blood glucose, and promoting weight loss (14, 56–58). In our study there was no significant change in Adipo-IR or blood glucose, but there was a decrease in body weight, suggesting that in cats FGF21 treatment did not significantly alter adipose insulin sensitivity or glucose disposal while still promoting lipolysis, suggesting that there are unique differences in the feline FGF21 pathway. Ideally, future studies should investigate adipose tissue stores from FGF21-treated and control cats and assess parameters including browning of adipocytes, FGF21 receptor distribution, inflammatory macrophage infiltration, and evaluation of markers of adipose tissue metabolism. Future non-invasive studies that evaluate FGF21 *in vivo* can investigate changes in circulating parameters such as obesity-associated inflammatory cytokines to indirectly assess adipose tissue inflammation (59), or determine if there are preferential sites of lipolysis in the adipose tissue [e.g., with computed tomography (CT)] (60). Determining if there is truly increased energy expenditure in cats, as suggested by the weight loss without increased caloric intake in our study, would require the use of metabolic cages adapted for cats (61).

While we observed substantial change in body weight, it is possible that the dose or treatment duration were not sufficient to produce a metabolic response. The dose and treatment duration were chosen based on published doses and time to parameter response without negative side effects for subcutaneous LY2405319 administrations in the published literature in obese humans with type 2 diabetes mellitus, type 2 diabetic rhesus macaques, and mice to determine the most likely effective dose and treatment period in cats. In obese T2DM humans, significant changes in metabolic parameters (serum glucose and triglycerides) were noted after 3–7 days at a dose of 3 mg per day (9). In rodents, subcutaneous administration of up to 1 mg/kg/day of LY2405319 resulted in decreased glucose by day 1 with a decreased plasma insulin following 7 days (12). In diabetic rhesus monkeys in a dose escalation study, there was a significant decrease in plasma glucose, triglycerides, and cholesterol by day 21 and significant changes were noted when a dose of 9 mg/kg was achieved (11). The duration of treatment in this study was also influenced by a study of streptozotocin-induced diabetic dogs subcutaneously administered 0.5 mg/kg/day of recombinant canine FGF21, which brought the blood glucose to levels close to those of the control non-diabetic dogs by day 4 (62). We selected a route of administration that would be easy for veterinary personnel to utilize in a future potential clinical setting. Subcutaneous administration is an extremely common route of administration in veterinary medicine and is achievable by veterinary personnel of many different expertise levels or even trained owners.

It may be possible that the lack of metabolic changes in this study reflect some of the inconsistent outcomes reported in the literature regarding various FGF21 analogs in animal models of insulin resistance and obesity. While decreases in blood glucose, insulin, plasma triglycerides, and low-density cholesterols and increases in high-density cholesterols were observed in T2DM non-human primates and humans treated with LY2405319 (9, 11), another FGF21 analog, PF-05231023, in contrast, had minimal effect on glucose and insulin in humans and monkeys (46). Although FGF21 is able to stimulate beneficial effects on a wide variety of tissues, the overwhelming majority of the glucose and adipose regulating functions of FGF21 appear to be via signaling on the liver and adipose tissue (14). In murine livers, FGF21 induces free fatty acid oxidation and increases glucose tolerance and energy expenditure. In adipose tissue, FGF21 induces browning and lipolysis and increases energy expenditure (14, 55). It is possible that the hepatic downstream signaling of FGF21 is diminished or altered in cats relative to the adipose tissue signaling, resulting in a decreased effect on insulin sensitivity compared to other animals.

One additional potential reason for the lack of metabolic changes is that LY2405319 is based on human FGF21 protein and not derived from the cat FGF21 protein. LY2405319 is a recombinant human FGF21 protein that had been engineered for increased *Pichia pastoris* host protein production and improved physical stability (12). The sequence chains of human FGF21 (UniProt identifier Q9NSA1 [29–209]) and feline FGF21 (M3W7L7 [28–208]) have an 84.5% identity and an 89.5% similarity using the EMBOSS Needle Pairwise Sequence Alignment tool (63), a slightly higher degree of structural similarity compared to human and mouse FGF21 (Q9JJN1 [29–210]), which have 80.5% identify and 84.9% similarity. As LY2404319 has similar pharmacological effects as FGF21 in mice (12), it reasons that LY2405319 would have similar pharmacological effects as endogenous FGF21 in cats due to the high protein similarity with humans. However, LY2405319 and feline FGF21 are not identical, and it is possible that these differences are enough to have reduced receptor activation and, consequently, the observed lack of metabolic results. The co-receptors of FGF21, beta-klotho (KLB) and fibroblast growth factor receptor 1 (FGFR1), have similarly relatively good interspecies conservation between cats and humans. Although no work, to the authors' knowledge, has been done on the feline FGF21 pathway machinery, it can be inferred from this homology that the signaling pathway is conserved. Feline KLB (M3XDH3) and human KLB (Q86Z14) have 83.8% identity and 90.3% similarity, and feline FGFR1 (A0A2I2UK29) and human FGFR1 (P11362) have 95.1% identity and 95.4% similarity. Investigations in gene and protein expression of FGF21, its receptors, and its downstream mediators in tissues, particularly the liver and adipose tissue, are warranted to determine which FGF21 pathways drive energy expenditure and glucose and lipid homeostasis in cats. In order to determine the cell-specific effects of the FGF21 pathway, additional studies are needed, particularly in primary feline hepatocyte and adipocyte cell cultures that investigate mechanisms such as adipokine and hepatokine secretion, lipolysis, and glucose disposal and consumption. Future potential studies such as these will allow us to investigate the physiologic responses in expected organ systems and help identify *in vitro* if species-specific differences in adipose or liver tissue signaling are contributing to the differences noted in cats (64, 65).

In our study, the change in liver triglyceride content based on 1H-MRS decreased in FGF21-treated cats and increased in the saline-treated cats, though these changes did not reach statistical significance. Concurrently, the change in ALKP was significantly decreased in FGF21-treated cats compared to the control cats. ALKP is a sensitive indicator of feline hepatic lipidosis (66) and based on our findings, we believe that a decrease in intrahepatic triglycerides may be driving these decreases in serum enzyme activity. These findings are particularly interesting as our obese cat colony does not have as marked an increase in hepatic triglyceride content compared to human and rodent studies. In our study, the baseline hepatic triglyceride content ranged from 2.71 to 12.19%. Studies investigating liver triglyceride content in obese non-human primates and humans report liver triglyceride contents consistently over 10% (67, 68), and in rodent studies, the hepatic triglyceride can reach near 90% (69). Changes in hepatic triglyceride content may be more pronounced in cats with hepatic pathology (e.g., lipidosis), and future studies are needed to determine if activation of the FGF21 pathway could be used to decrease hepatic triglyceride in more severely affected cats. There is some evidence that FGF21 can be used as an anti-inflammatory in chronic pancreatitis (70, 71). As a large proportion of cats with pancreatitis also have hepatic lipidosis (72, 73), further investigation of the anti-inflammatory effects of FGF21 analogs in cats with pancreatitis and concurrent hepatic lipidosis is needed, particularly since there are very few effective therapeutic options for these conditions, and access to medications that can reduce liver lipid content and reduce pancreatic inflammation could have an important impact on reducing the morbidity and mortality of hospitalized feline patients.

Compared to their baseline values, FGF21 treatment decreased serum AST activity on Day 14; however, there was no difference in the change in AST activity between saline-treated and FGF21-treated cats. A concurrent decrease in the hepatocellular leakage enzyme ALT was not observed and suggested that non-hepatic sources of AST activity, such as in the muscle (66, 74), could contribute to this finding. In mice, FGF21 acts as a myokine and is associated with decreased muscle mass and impaired mitochondrial function, but the biological significance of these findings is debatable due to low muscle receptor expression (75, 76). Investigations into FGF21 gene and receptor expression within skeletal muscle in cats may be needed to determine the effects of FGF21 on feline skeletal muscle.

There are limited studies that investigate the effects of FGF21 on the gut microbiome (17), and evidence suggests that the hepatic FGF21 adaptive metabolic response is mediated by the gut microbiome (16). We did not note any significant difference in microbial diversity between treated and control cats, and none of the eight thousand bacterial species showed significant change in abundance at a 5% FDR. If a less stringent statistical cut-off of 10% FDR was applied, 246 bacteria showed alterations in relative abundance. However, all but one had extremely low abundance (<0.01%), which is likely to reflect sampling variability rather than biological relevance. Concurrent with our results, a recent study in humans administered FGF21 also noted that treatment did not affect fecal microbiome taxonomy (17). Recently, the metagenomic sequence of the feline gut microbiome was described, and distinct differences in the diversity and abundance of certain species were noted between lean and obese cats (13). While the cats in our study did lose significant weight, they did not lose enough weight to significantly reduce their body condition score or to reclassify them as having a healthy body weight. In this study, FGF21 was administered subcutaneously, meaning that effects, if any, would likely be associated with the ability of FGF21 to alter the host environment. It is also likely that the microbiome may alter the FGF21 adaptive stress response, but not the other way around.

Elastography measures the stiffness or viscosity of an organ and has been proposed as a point of care, non-invasive method to evaluate chronic liver diseases in humans, including the most common cause of liver disease in people, non-alcoholic fatty liver disease. In our study, there were no changes in hepatic elasticity before or after treatment, and there was no difference in the change of elasticity between treatment groups, consistent with other recent studies (77). For humans, shear wave elastography correlates well with fibrosis in liver biopsies, the current gold standard (78). Unlike feline chronic lymphocytic cholangiohepatitis (79) or congenital hepatic fibrosis (80), feline hepatic lipidosis is a more acute disease syndrome that does not involve substantial fibrosis or cirrhosis. As such, an additional goal of our study was to determine if liver elasticity would be affected by subtle changes in the liver lipid content associated with FGF21. Based on our findings, it is unlikely that elastography is sensitive enough to detect changes associated with lipid accumulation and thus has little potential to be used as a less invasive point-of-care monitoring tool for feline hepatic triglyceride content, but further studies, including the use of other modalities of elastography, are needed to evaluate the clinical use of elastography in chronic veterinary hepatic diseases, particularly those with prominent fibrosis. Additional diagnostic tools to better quantify liver lipid content rapidly and effectively could provide greater insight into hepatic health. It would also be interesting to investigate changes in liver elasticity in animals with more severe hepatic lipid accumulation, such as hepatic lipidosis.

One major limitation of our study was the number of cats in each group, which was limited by the number of obese and overweight cats within the feline obesity research colony. This likely led to an under powering of many of the metabolic and liver parameters. Despite this under powering, the FGF21 treated group had a significantly increased rate of weight loss compared to the control group indicating a significant drug effect. It would be worthwhile to expand FGF21 treatment to a larger cohort of cats, potentially client-owned, and evaluate if there are decreases in liver lipid reduction. It is possible that repeating the study with a longer treatment period may yield more dramatic changes. Additional studies are needed to supplement these pilot data in order to fully explore the pharmacokinetics of this FGF21 mimetic in cats, particularly if there are potential therapeutic benefits to cats.

In summary, activation of the FGF21 pathway can safely induce weight loss with trends to improve liver lipid content in obese and overweight cats. Under these study conditions, FGF21 does not appear to alter glucose homeostasis or serum lipids in cats with mild metabolic dyscrasias. Independent of lipid and glucose-modulating effects, manipulation of the FGF21 pathway may potentially be therapeutic for feline obesity and more importantly hepatic lipidosis. This is the first study investigating an FGF21 analog in cats and demonstrates a potential unique metabolic response. Further work is needed to better understand the feline FGF21 signaling pathway.

## Data availability statement

The whole-genome shotgun metagenomic sequencing data presented in the study are deposited in the NCBI Short-Read Archive repository, under accession numbers PRJNA758898 and PRJNA821230.

## Ethics statement

The animal study was reviewed and approved by Auburn University's Institutional Animal Care and Use Committee (IACUC) with protocol number 2019-3482.

## Author contributions

EG and EB were responsible for the conception and primary design of the study. EB, EG, AB, RC, and XW contributed to the design of the study. EB, TT, RW, AB, RC, XM, XW, and EG all contributed to data collection. EB performed the statistical analysis and wrote the first draft of the manuscript. EB, EG, AB, XW, XM, and RC wrote sections of the manuscript. All authors contributed to manuscript revision, read, and approved the submitted version.

## Supplementary material

Supplementary Figure S1 Total weight, water intake and food intake of cats over the study period. On day 14 (#), cats were held without food overnight in preparation for general anesthesia and 1H-MRS data. Shaded areas between days 1 and 14 represent the period where either FGF21 or saline vehicle were given.

Supplementary Figure S2 Circulating metabolic parameters. **(A)** Baseline blood glucose was similar between treatment groups, although the blood glucose concentration in the FGF21-treated cats was 29.1% higher (49.1 mg/mL) than the saline blood glucose concentration. **(B)** In the FGF21-treated group, serum insulin decreased 25.5% from 40.08 ng/mL to 29.86 ng/mL. **(C)** In the FGF21-treated group, HOMA-IR decreased 22.9% from 3.55 to 2.74. In the control group, HOMA-IR decreased 35.0% from 2.59 to 1.68. **(D)** In the FGF21-treated group, NEFAs increased 46.8% from a mean of 0.23 mEq/L to 0.34 mEq/L. In the control group, NEFAs increased 24.9% from 0.25 mEq/L to 0.31 mEq/Le. **(E)** Serum triglycerides in the control group decreased 35.1% from 51.33 mg/dL to 33.33 mg/dL, and decreased 34.4% in the FGF21 treated group from 32.0 mg/dL to 21.0 mg/dL. **(F)** Serum cholesterol in the control group rose 0.20% from 167.3 mg/dL to 167.7 mg/dL, and serum cholesterol in the FGF21-treated group decreased 3.72% from 161.5 mg/dL to 155.5 mg/dL. **(G)** Treatment with FGF21 did not alter Adipo-IR. **(H)** In the FGF21-treated group, Adipo-IR increased 1.27% from 1.728 to 1.75. Adipo-IR in the control group remained at a mean of 1.60.

Supplementary Figure S3 FGF21 tends to lower liver lipid stores but does not alter liver tissue stiffness. **(A)** The fraction liver lipid measured by 1H-MRS is not significantly altered by FGF21 (paired *t*-test). **(B)** In the control group, liver elasticity decreased 26.4%, from 12.6 kPa to 9.25 kPa, while liver elasticity in the FGF21 group increased 4.25%, from 8.63 kPa to 9.0 kPa with treatment.

Supplementary Figure S4 Liver serum biomarker changes are consistent with decreased liver lipid content. **(A)** In the control group, ALKP increased 8.4% during the treatment period, while ALKP activity in the FGF21-treated group decreased 24.8% from a mean of 37.25 U/L to 28.00 U/L. **(B)** ALT and **(C)** Total Bilirubin are not altered by FGF21 treatment. **(D)** There was a 23.4% decrease in the enzyme aspartate aminotransferase (AST) in the FGF21-treated cats, from 31 U/L to 23.75 UL. The control cats had a 1.43% increase in AST, from 23 U/L to 23.3 U/L.
