# Supplementary figures and images for: Direct activation of the fibroblast growth factor-21 pathway in overweight and obese cats
Source: Front Vet Sci. 2023 Jan 23;10:1072680. doi: 10.3389/fvets.2023.1072680 (PMC9900002; doi:10.3389/fvets.2023.1072680)

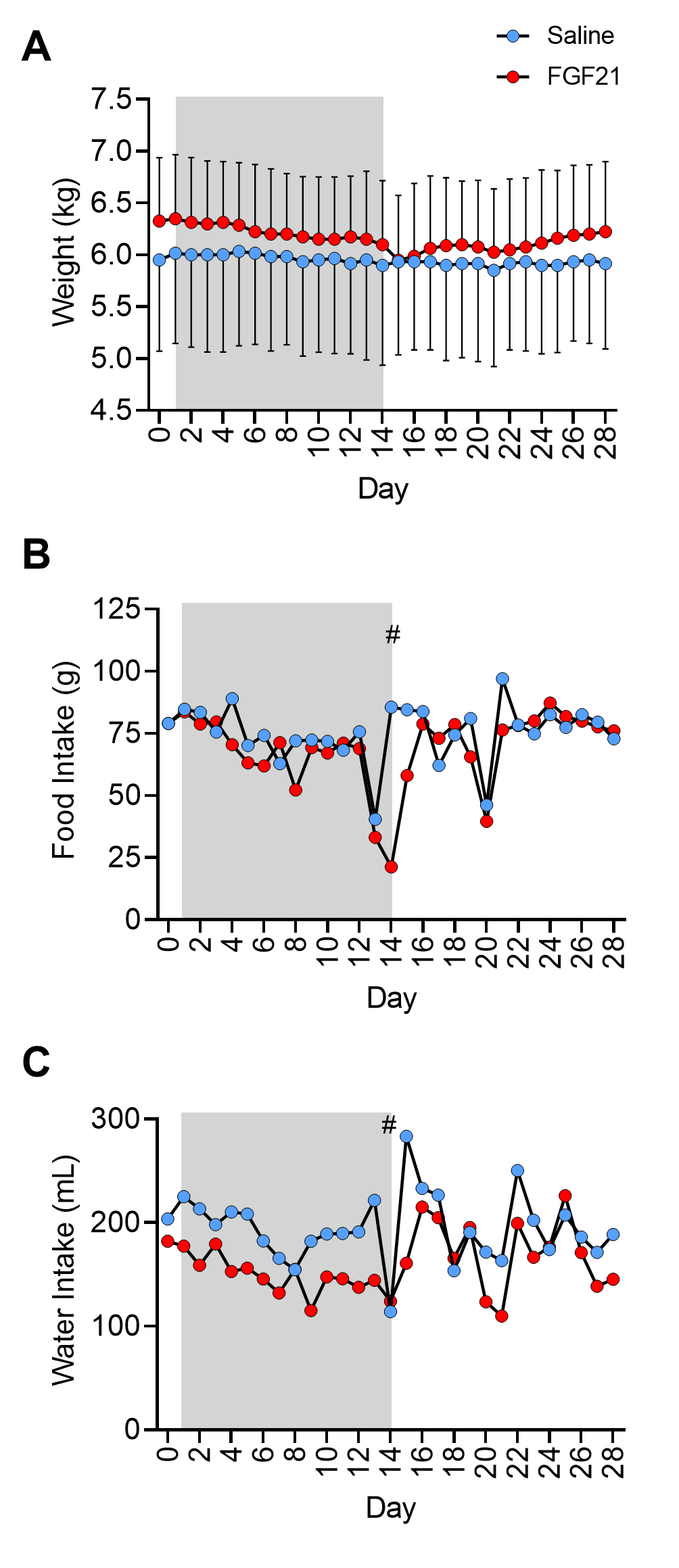

Supplement: Supplementary Figure S1 — Total weight, water intake and food intake of cats over the study period. On day 14 (#), cats were held without food overnight in preparation for general anesthesia and 1H-MRS data. Shaded areas between days 1 and 14 represent the period where either FGF21 or saline vehicle were given. [file Image_1.TIF]

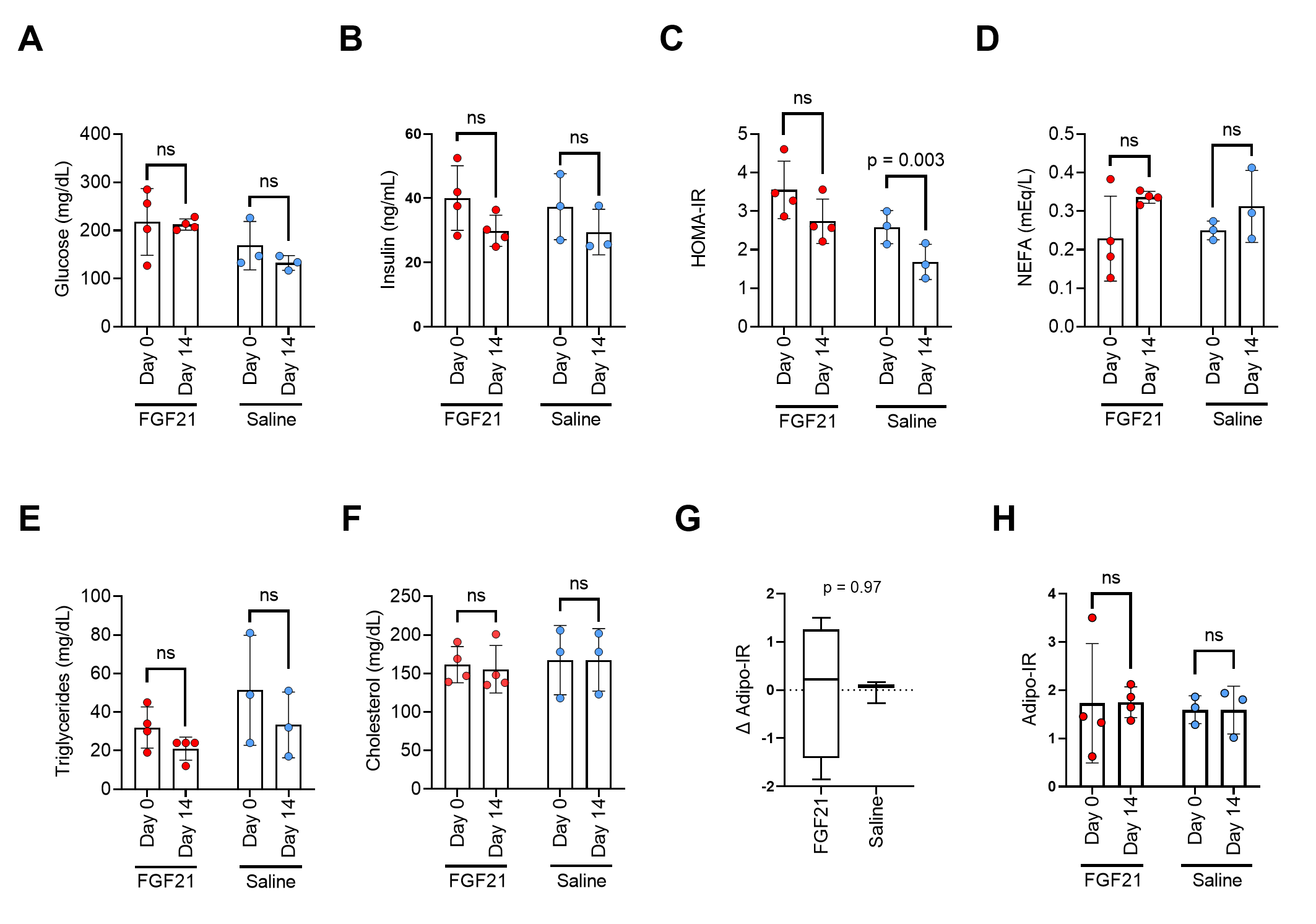

Supplement: Supplementary Figure S2 — Circulating metabolic parameters. (A) Baseline blood glucose was similar between treatment groups, although the blood glucose concentration in the FGF21-treated cats was 29.1% higher (49.1 mg/mL) than the saline blood glucose concentration. (B) In the FGF21-treated group, serum insulin decreased 25.5% from 40.08 ng/mL to 29.86 ng/mL. (C) In the FGF21-treated group, HOMA-IR decreased 22.9% from 3.55 to 2.74. In the control group, HOMA-IR decreased 35.0% from 2.59 to 1.68. (D) In the FGF21-treated group, NEFAs increased 46.8% from a mean of 0.23 mEq/L to 0.34 mEq/L. In the control group, NEFAs increased 24.9% from 0.25 mEq/L to 0.31 mEq/Le. (E) Serum triglycerides in the control group decreased 35.1% from 51.33 mg/dL to 33.33 mg/dL, and decreased 34.4% in the FGF21 treated group from 32.0 mg/dL to 21.0 mg/dL. (F) Serum cholesterol in the control group rose 0.20% from 167.3 mg/dL to 167.7 mg/dL, and serum cholesterol in the FGF21-treated group decreased 3.72% from 161.5 mg/dL to 155.5 mg/dL. (G) Treatment with FGF21 did not alter Adipo-IR. (H) In the FGF21-treated group, Adipo-IR increased 1.27% from 1.728 to 1.75. Adipo-IR in the control group remained at a mean of 1.60. [file Image_2.TIF]

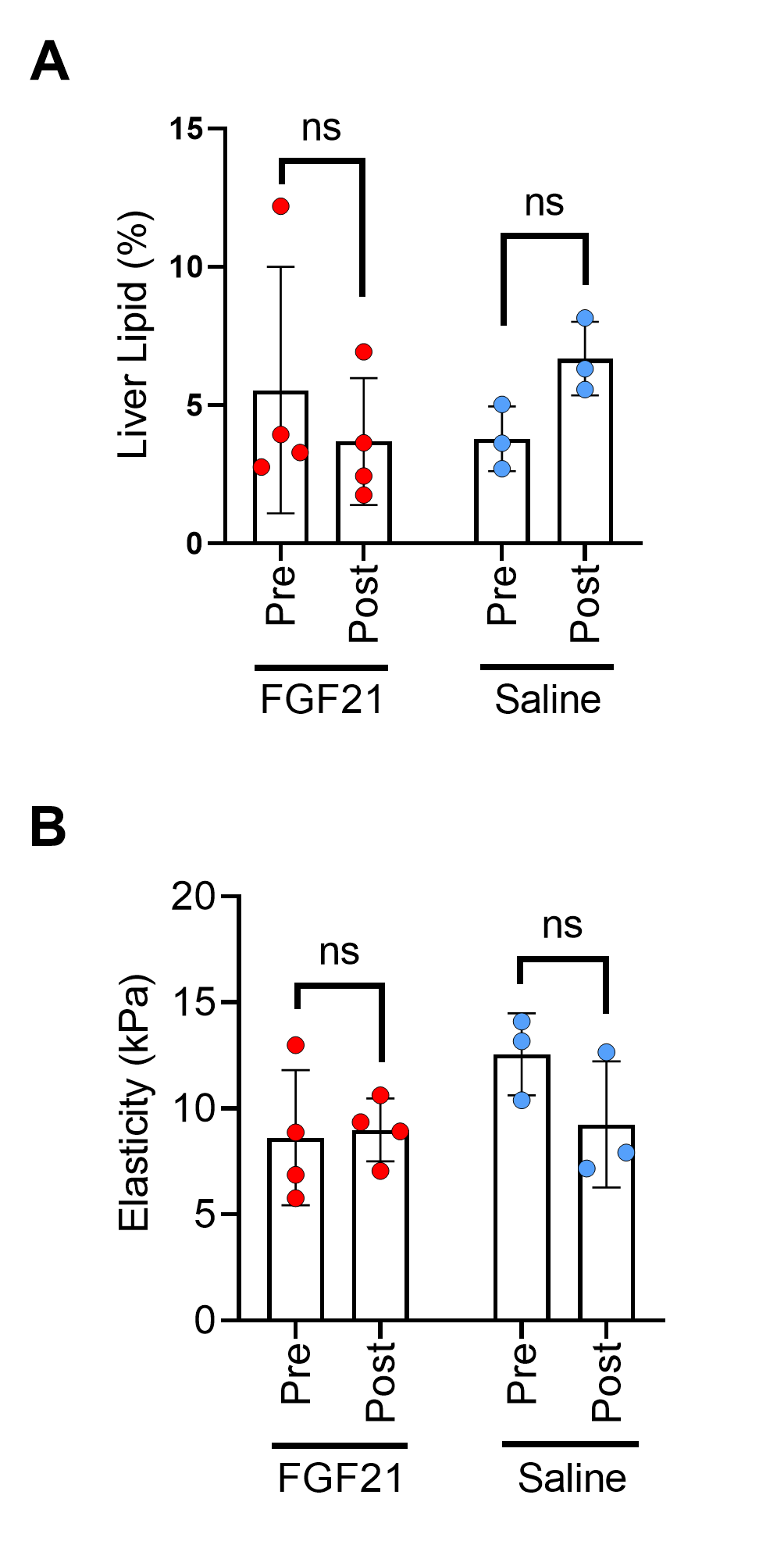

Supplement: Supplementary Figure S3 — FGF21 tends to lower liver lipid stores but does not alter liver tissue stiffness. (A) The fraction liver lipid measured by 1H-MRS is not significantly altered by FGF21 (paired t-test). (B) In the control group, liver elasticity decreased 26.4%, from 12.6 kPa to 9.25 kPa, while liver elasticity in the FGF21 group increased 4.25%, from 8.63 kPa to 9.0 kPa with treatment. [file Image_3.TIF]

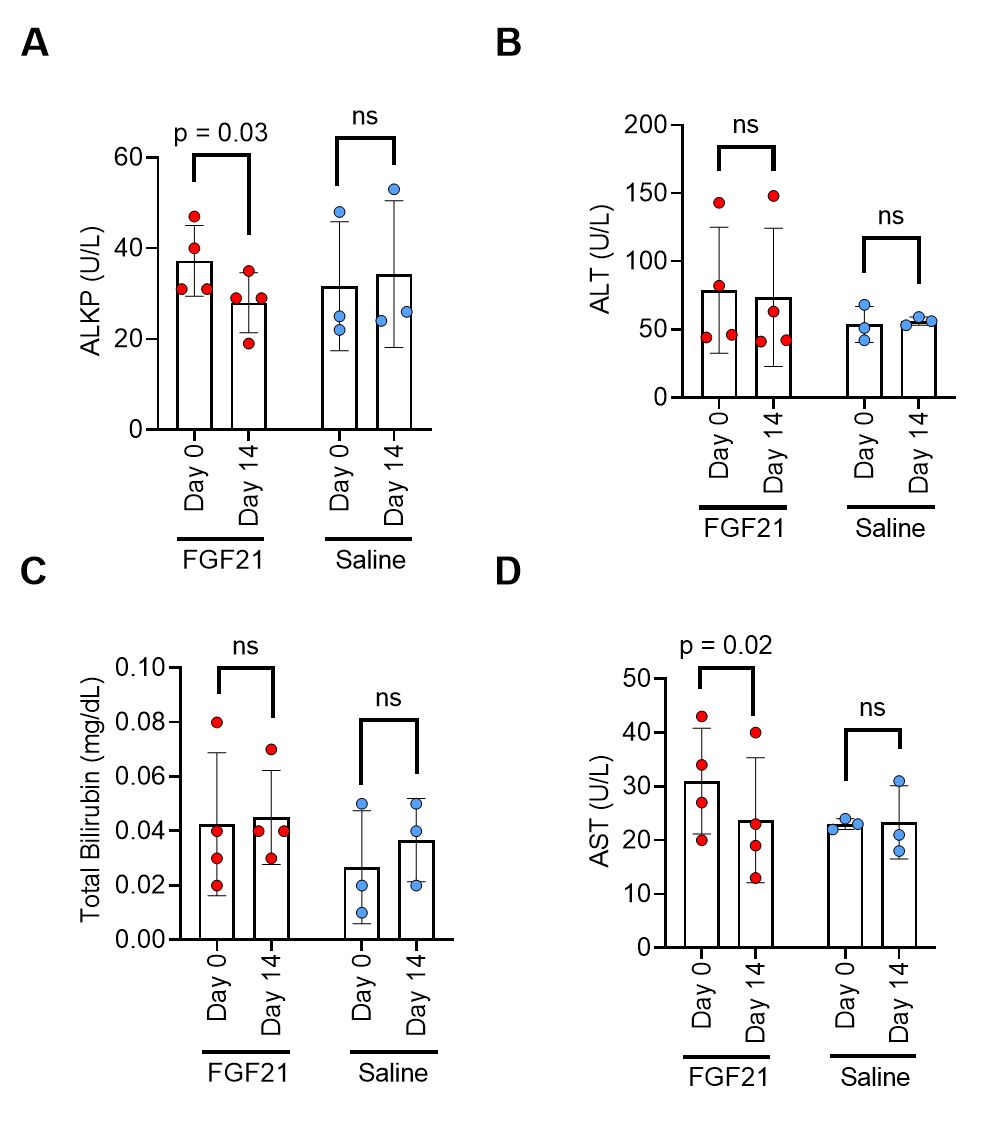

Supplement: Supplementary Figure S4 — Liver serum biomarker changes are consistent with decreased liver lipid content. (A) In the control group, ALKP increased 8.4% during the treatment period, while ALKP activity in the FGF21-treated group decreased 24.8% from a mean of 37.25 U/L to 28.00 U/L. (B) ALT and (C) Total Bilirubin are not altered by FGF21 treatment. (D) There was a 23.4% decrease in the enzyme aspartate aminotransferase (AST) in the FGF21-treated cats, from 31 U/L to 23.75 UL. The control cats had a 1.43% increase in AST, from 23 U/L to 23.3 U/L. [file Image_4.TIF]
