# Supplementary material for: Direct activation of the fibroblast growth factor-21 pathway in overweight and obese cats
Source: Front Vet Sci. 2023 Jan 23;10:1072680. doi: 10.3389/fvets.2023.1072680 (PMC9900002; doi:10.3389/fvets.2023.1072680)
Supplement: Supplementary file 5 [file Table_1.DOCX]

**Table S1. Whole genome shotgun metagenomic sequencing yield, control statistics, and accession IDs.**

| **cat ID** | **Treatment** | **Sample Number** | **Accession ID** | **# of reads** | **Sequences (GB)** | **host contamination %** |
| --- | --- | --- | --- | --- | --- | --- |
| D001 | FGF21 | 1 | SRR18550130 | 86,166,510 | 12.9 | 1.08% |
|  |  | 2 | SRR18550144 | 96,593,290 | 14.5 | 0.96% |
| F001 | FGF21 | 1 | SRR18550129 | 101,557,634 | 15.2 | 31.78% |
|  |  | 2 | SRR18550143 | 123,360,100 | 18.5 | 22.24% |
| H001 | FGF21 | 1 | SRR18550141 | 112,269,038 | 16.8 | 16.29% |
|  |  | 2 | SRR18550135 | 43,897,220 | 6.6 | 6.34% |
| K001 | FGF21 | 1 | SRR18550138 | 91,228,332 | 13.7 | 6.59% |
|  |  | 2 | SRR18550132 | 23,219,858 | 3.5 | 25.47% |
| I001 | saline | 1 | SRR18550140 | 84,738,496 | 12.7 | 8.73% |
|  |  | 2 | SRR18550134 | 42,586,664 | 6.4 | 17.24% |
| G001 | saline | 1 | SRR18550142 | 86,937,904 | 13.0 | 1.16% |
|  |  | 2 | SRR18550136 | 72,136,664 | 10.8 | 2.73% |
| J001 | saline | 1 | SRR18550139 | 72,200,540 | 10.8 | 3.12% |
|  |  | 2 | SRR18550133 | 81,111,494 | 12.2 | 4.04% |
