# Supplementary material for: Direct activation of the fibroblast growth factor-21 pathway in overweight and obese cats
Source: Front Vet Sci. 2023 Jan 23;10:1072680. doi: 10.3389/fvets.2023.1072680 (PMC9900002; doi:10.3389/fvets.2023.1072680)
Supplement: Supplementary file 6 [file Table_2.docx]

**Table S2**. Weights of each cat assigned to either the FGF21-treated or control group taken take baseline (Day 0), post-treatment period (Day 15), and post-washout period (Day 28). Each row represents an individual cat.

|  | **Baseline weight (kg)** | **Post-treatment weight (kg)** | **Post-washout period weight (kg)** |
| --- | --- | --- | --- |
| FGF21-treated cats | 6.95 | 6.60 | 6.90 |
|  | 5.50 | 5.10 | 5.30 |
|  | 6.55 | 6.10 | 6.45 |
|  | 6.30 | 6.00 | 6.25 |
| Control cats | 5.30 | 5.25 | 5.30 |
|  | 6.95 | 6.95 | 6.85 |
|  | 5.60 | 5.60 | 5.60 |
